# Desk based prompts to replace workplace sitting with stair climbing; a pilot study of acceptability, effects on behaviour and disease risk factors

**DOI:** 10.1186/s12889-022-14393-1

**Published:** 2022-10-31

**Authors:** Intan Suhana Munira Mat Azmi, Gareth A. Wallis, Mike J. White, Anna Puig-Ribera, Frank F. Eves

**Affiliations:** 1 School of Sports, Exercise and Rehabilitation Sciences, College of Life and Environmental Sciences, University of Birmingham, Birmingham, UK; 2 Community Medicine Unit, Faculty of Medicine, Universiti Sultan Zainal Abidin (UniSZA), Medical Campus, Jalan Sultan Mahmud, 20400 Kuala Terengganu, Terengganu Malaysia; 3 Centre for Health and Social Care Research, Department of Physical Activity Sciences, Universitat de Vic-Universitat Central de Catalunya, Barcelona, Spain

**Keywords:** Prolonged sitting, Workplace, Point-of-choice prompts, Stair climbing, Acceptability, Serum lipids, Glucose

## Abstract

**Background:**

Prolonged sitting is associated with increased risk of obesity, type 2 diabetes and cardiovascular disease. Occupational sitting accounts for up to 50 h/week for employees. This pilot study assessed the acceptability of stair climbing as an interruption to sitting throughout working hours, and provided preliminary data of the effects on glucose and lipid profiles.

**Methods:**

A quasi-experimental design was conducted involving 16 sedentary office workers (five females and 11 males) for intervention (n = 8) and control groups (n = 8) with mean age of 36.38 (5.58). For the eight-week intervention, a continuous four-floor stair climb and descent was performed eight times/day spread evenly over the working day. A prompt to climb was presented on the participant’s computer eight times/day. Participants in the experimental group recorded daily floors climbed and steps (measured using pedometers) in a weekly log sheet. Blood samples were collected pre and post intervention to test effects on fasting glucose and 2 h plasma glucose, triglycerides, and total (TC), LDL and HDL cholesterol. Experimental participants were interviewed at the end of the study. The Wilcoxon signed rank test was used to compare the median changes (pre-post) of the dependent variables.

**Results:**

On average, the experimental group climbed 121 floors/week when prompted. There were significant reductions in fasting blood glucose, TC and LDL, as well as the derived measures of ‘bad’ cholesterol and the TC/HDL ratio in the experimental group. Post-experimental interviews indicated that the interruption to sitting was well tolerated.

**Conclusion:**

Prompted stair climbing activity had impacts on health outcomes and was found acceptable to employees at work.

**Trial registration:**

Ethics for this study was approved by Science, Technology, Engineering and Mathematics Ethical Review Committee, University of Birmingham with ethics reference number ERN_15_0491.

**Supplementary information:**

The online version contains supplementary material available at 10.1186/s12889-022-14393-1.

## Background

The Metabolic Syndrome (MetS) is a cluster of physiological markers that increases the risk of cardiovascular disease and all-cause mortality [1]. Elevated abdominal adiposity, high triglyceride levels, low levels of high density lipoprotein cholesterol (HDL-C), elevated blood pressure, and high levels of fasting blood glucose define MetS [2]. Sedentary behaviour is a risk factor for MetS, with effects independent of physical activity level [3].

Sedentary behaviour is defined as any waking behaviour in a supine, seated, or reclined posture that expends less than or equal to 1.5 metabolic equivalent (METs) [4, 5]. Sedentary behaviour frequently occurs when working in the office, commuting, and spending leisure time on screen based media such as television, video games and a host of internet pastimes [6]. Sitting is a ‘routine’ of work and can be described as accidentally unhealthy. Often individuals are unaware that they are sitting, reporting that it occurs as part of a work-related task [7] e.g. prolonged sitting at desk in front of a computer, long hours of meetings and seminars and continuous telephone answering throughout the working period. Thus, workplace sitting is primarily a non-conscious behaviour that facilitates the over-arching goal of working.

Interventions to counteract prolonged sitting at work have trialled sit-to-stand desks to replace sitting with standing [8–10]. While substantial reductions in sitting were achieved in all three studies, only modest improvements in health markers were reported [8, 9]. Importantly, these studies replaced sitting at ≤ 1.5 METs primarily with the low intensity physical activity of standing, 1.8-2.0 METs [11]. Both cross-sectional and prospective observational data indicate that physical activity at any intensity, either moderate or vigorous to break sedentary time is associated with reduced risk of MetS although vigorous physical activity is the most beneficial [12–15]. Vigorous interruption to sitting is available in workplaces, namely stair climbing. Climbing stairs continuously requires 8.6 METs, even at a slow pace [16], and 9.6 METs when climbing at a typical pace [17]. Even short bouts of stair use will have beneficial effects [18, 19].

One intervention approach that interrupts routine behaviour is point-of-choice prompting [20, 21]. Previous research used point-of-choice prompting with simple health-promoting messages, e.g. ‘*stair climbing burns more calories per minute than jogging*’, prompted shoppers and commuters to climb the stairs, thus breaking their routine of taking the escalator [20, 22]. A number of successful studies have adapted prompting to reduce workplace sitting [10, 23–25]. All added an educational component about the potential benefit of reducing sedentary behaviour, i.e. multi-component interventions. In one study, the prompt was 4.8 times more effective than no-prompt, emphasising the routine nature of the behaviour that needs interrupting [23]. While all studies were successful, the behaviour which replaced sitting was unreported. However, Cooley and co-worker stated that the three most popular choices from 60 potential behaviours were stair climbing, walking and chair squats [23]. Stair climbing is a plausible interruption to sitting for employees. Consistent with this, star climbing was viewed generally by employees as beneficial for cardiovascular health and a behaviour that would make them more alert and motivated at work [26]. While stair climbing had comprehensive effects on MetS risk with a sufficient sample size [27], encouraging effects on serum lipids have been reported with relatively small samples (n = 12, [28]; n = 8, [29]).

This study introduced a stair climbing regime that has not been tested on its health effects towards sedentary office workers. Climbing four floors requires two minutes to complete and provides short bouts of vigorous physical activity in breaking the sedentary behaviour at work. Thus, the aims of this pilot study were to observe the effect of the prompt in increasing stair climbing activity, and if a four-floor climb would be acceptable to the employees. This study also aimed to assess any improvements that might occur in glucose and lipid profiles.

## Methods

### Study sites

The study was conducted at the Biosciences (eight floors) and Muirhead Tower (12 floors) buildings, University of Birmingham. Both sites were more than four-storey buildings to suit the stair climbing activity of the intervention. Participants in the experimental group worked at one of these buildings. The staircases were located near to the participants’ office to facilitate its use eight times/day. Permission to conduct the study and install prompting software on office computers had to be obtained from each building management, in particular the IT section. In addition, we were precluded from recruiting employees working on financial tasks as the building management were concerned that the prompt might introduce errors by interrupting behaviour during an important calculation. The control group consisted of participants that worked at other buildings which were less than four storeys. The pre and post intervention visits were held in Human Performance Laboratory (HPL) at School of Sport, Exercise & Rehabilitation Sciences, University of Birmingham.

### Study population

Posters with the title “*Can you spare 16 minutes a day at work to get healthier?*” were put up on the notice board or affixed to the wall adjacent to the lift in the buildings. We hoped that the strapline with the word *‘healthier’* would recruit individuals who were motivated to improve their health and, hence, increase their physical activity. Inclusion criteria were (a) office workers at the University of Birmingham with a primarily sedentary job, (b) non-smoker, (c) able to climb stairs regularly, and (d) healthy and not on any medications. Participants (n = 16) were allocated to experimental and control groups using self-selection sampling method. One experimental participant could not complete the intervention and was replaced. The dropout said she was *‘unable to commit due to her work position that involved a number of long meetings on most of the working days’*. The study started on 17th August 2015 and ended on 2nd December 2016, with the last completed participant for the experimental group. All participants gave informed consent prior to enrolment. Throughout the study, participants were asked to maintain their customary physical activity and diet.

### Behavioural measures

Each experimental participant wore a pedometer throughout the study to allow a preliminary test of whether any increases in stair climbing were associated with a compensatory reduction in the number of steps during the week. At the end of each week, the number of steps was read from the pedometer by the experimenter. In addition, a log sheet with time-slot for the eight prompts on each day was provided so that participants could record the number of flights climbed in response to each prompt.

### Study protocol

- Pre-intervention visit

Participants fasted overnight from 9 pm prior to the pre-intervention visit in the morning. During this visit, height and weight were measured prior to the oral glucose tolerance test (OGTT) and a cannula inserted into the antecubital vein for blood sampling. A 10 ml sample was taken before the consumption of the concentrated sugar drink (75 g dextrose powder in 300 ml of water) to provide a baseline level. Then, blood sampling (5 ml) was performed at 30-minute intervals until two hours after the beverage was consumed (30, 60, 90, & 120 min) [30]. The cannula was irrigated every 15 min with 0.9% sodium chloride to avoid blood coagulation.

b) Experimental intervention

Participants in the experimental group were instructed to climb stairs daily as brief interruptions to their sedentary time at work. Participants were instructed to climb four floors of stairs continuously, equalling 14 m of climb eight times/day, and then descend by the stairs which summed to 32 floors/day. Climbing four floors was estimated to take about 60 s with a further 60 s to descend after the climb.

A prompt to climb was provided by participant’s desktop to climb at the appointed time using the software called task scheduler. The message ‘*It’s time for a break. Please, climb the stairs for your health.*’ appeared at the bottom right corner of the screen. These prompts were spread evenly throughout the day during participant’s working hours, based on an eight-hour working day. Participants were required to complete the log sheet after each prompt to log the number of floors of each climb. The intervention lasted eight weeks. Participants in the control group were asked to continue with their daily routine as usual.

c) Post-intervention visit

Procedures during the pre-intervention visit were repeated. In addition, experimental participants were interviewed by the researcher about their experience of the stair climbing intervention. They were asked to respond in their own words to the questions ‘*Why did you volunteer for this study?*’, ‘*Was the stair climbing intervention convenient/easy for you?*’, ‘*Did the intervention help or interrupt with your work?*’, ‘*Are you going to continue climbing stairs regularly?*’, ‘*Would you participate in this sort of intervention again?*’, ‘*Can you think of any benefits from this intervention?*’, ‘*Can you think of any barriers to this intervention?*’, ‘*What would you like to improve from this activity?*’, and ‘*What did your colleagues say about it?*’. The duration of the interview was approximately 10 min and took place in the laboratory after post-intervention blood sampling procedure. The responses were recorded verbatim.

d) Laboratory analyses

Collected venous blood was dispensed into serum tubes and left to clot at room temperature for 30 min prior to centrifugation at 3000 rev.min^− 1^ for 15 min. Aliquots containing serum were stored at -20˚C until analysis. Sample analysis was performed in duplicate using enzymatic colorimetric assays for serum glucose, triglycerides (TG), total cholesterol (TC), high density lipoprotein cholesterol (HDL-C) and low density lipoprotein cholesterol (LDL-C) using an ILAB 650 clinical chemistry analyser (all Instrumentation Laboratories, Cheshire, UK). In addition, the TC/HDL-C ratio and non-HDL-C (TC- HDL-C) were calculated.

### Statistical analysis

Data were analysed using the SPSS statistical package for Windows, version 20.0. Data did not meet the assumptions for parametric statistical analysis due to the small sample size and data distributions showed skewness when tested for normality. Thus, non-parametric tests were used and median with interquartile range (IqR) were reported instead of mean and standard deviation (SD). The Wilcoxon signed rank test was used to compare the median changes (pre-post) of fasting glucose, response to the OGTT, TG, TC, LDL-C, HDL-C, TC/HDL-C ratio and non-HDL-C for control and experimental groups. In this study, participants were asked to climb four floors, eight times/day, a less extensive climb than the eight floors used in the study by Boreham and colleagues [28]. For all these analyses, we predicted reduced concentrations post vs. pre for the experimental group but not for the control group with the exception of the opposite effect for HDL-C. As this was a pilot study with some physiological measures, the analyses of the physiological data did not adopt a conventional approach but rather tested the best-case scenario with this relatively small sample that was underpowered for effects of the less intense intervention than that employed by Boreham and colleagues. Nonetheless, the separate group analyses pre/post matched those reported by Boreham et al. (2000) [28].

For the data related to changes over weeks in the number of floors climbed and pedometer steps for the experimental group, analyses employed repeated measures analyses of variance, with the single degree of freedom linear, quadratic and cubic polynomials. These analyses tested for any change over weeks during the intervention. Any changes in the number of floors climbed could indicate adaptation to the intervention. For pedometer steps, the analyses tested for possible replacement of steps with stair use.

## Results

The table below displays the descriptive statistics of age and BMI for each group (Table 1). Comparisons between the groups revealed that the age of the experimental group was higher than that of the control group. Both groups were overweight.

**Table 1:** Participants’ characteristics at baseline

Variables	Control (*n* = 8)	Experimental (*n* = 8)
	**Mean (** ***SD*** **)**	**Mean (** ***SD*** **)**
Age (years)	36.38 (5.58)	44.75 (12.56)
BMI	29.70 (9.21)	30.78 (6.27)

### Physiological effects of the intervention

Table 2 summarises the median (IqR) for pre and post for both control and experimental groups. As can be seen from the table, there were no significant changes in the control group pre vs. post. In contrast, there were improvements in LDL-C, TC, TC/HDL-C ratio, non-HDL-C and fasting blood glucose in the experimental group. As noted in the methods section, these analyses represent the best-case scenario. Replication with a larger sample, and, hence, greater statistical power, would be required to confirm the effects relative to a control group.

**Table 2:** Median (IqR) for the study variables pre and post for the control and experimental groups, with a summary of statistical testing

Variables (mmol.L^− 1^)	Control Group (n = 8)		Control x Pre:post	Experimental Group (n = 8)		Exp^a^ x Pre:post
	**Pre Median (IqR)**	**Post Median (IqR)**	**Z statistic** ***p*** **value**	**Pre Median (IqR)**	**Post Median (IqR)**	**Z** **statistic** ***p*** **value**
TG	1.52 (1.01)	1.40 (0.87)	− 0.70 *p* = .49	1.53 (1.00)	1.34 (1.06)	-1.12 *p* = .26
TC	5.35 (1.25)	5.30 (1.26)	-1.48 *p* = .13	5.00 (2.08)	4.70 (2.00)	**-2.38** ^**c**^ ***p*** **= .02**
HDL-C	1.11 (0.33)	1.06 (0.30)	-1.36 *p* = .17	1.11 (0.58)	1.22 (0.59)	0.35 *p* = .73
LDL-C	3.50 (1.08)	3.50 (1.14)	-1.33 *p* = .18	3.20 (1.53)	3.05 (1.28)	**-2.20** ***p*** **= .03**
TC/HDL-C ratio	4.94 (1.34)	4.98 (1.69)	-0.28 *p* = .78	5.30 (1.44)	4.58 (1.49)	**-2.52** ***p*** **= .01**
non-HDL-C	4.14 (1.21)	4.11 (1.09)	-1.12 *p* = .26	3.90 (1.43)	3.64 (1.49)	**-2.52** ***p*** **= .01**
Fasting glucose	5.13 (1.09)	5.09 (1.19)	0.42 *p* = .67	5.14 (0.77)	4.93 (0.55)	**-2.24** ***p*** **= .03**
OGTT AUC^b^	6.22 (1.97)	6.70 (1.13)	− 0.14 *p* = .89	7.63 (1.99)	7.26 (0.95)	-1.40 *p* = .16

^a^ Exp = experimental. ^b^ OGTT AUC = oral glucose tolerance test, area under the curve. ^c^ Significant changes pre vs. post are presented in bold

### Behavioural response to the intervention

**Fig. 1:**
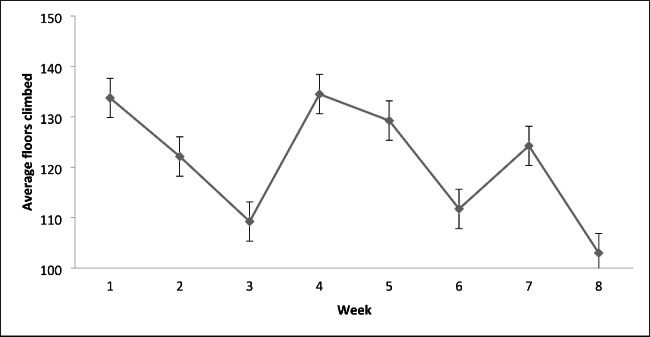
Average number of self-reported climbs each week by experimental group

Figure 1 below depicts the average number of floors climbed in response to the prompt. The floors climbed data were self-reported. In analyses of the number of floors climbed, there was no main effect of week, *F*_(4, 25.1)_ = 1.82, *p* = .16, after applying the Greenhouse-Geiser correction to the degrees of freedom (Epsilon = 0.512). The linear, quadratic and cubic polynomial trends were all non-significant (all prob > 0.18). These data do not suggest any incremental effects on the number of floors climbed over the period of the intervention. Instead, there is some variation over weeks (mean floors climbed) that reflect individual variability in response to the prompt (Fig. 1). On average, the experimental group climbed 121 (20) floors/week equivalent to 3388 m of climbing over the duration of the experiment.

**Fig. 2:**
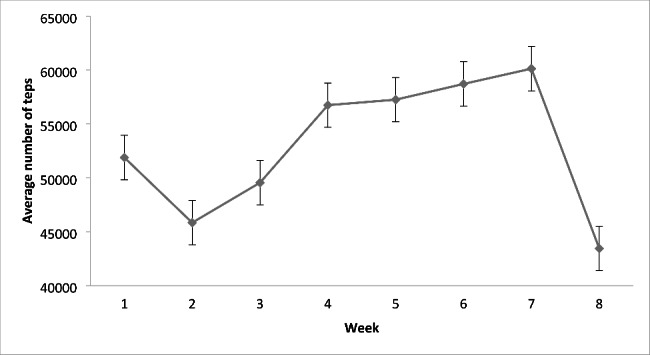
Average number of pedometer steps measured each week in the experimental group

Figure 2 depicts the number of pedometer measured steps each week in the experimental group. In analyses, there was a significant effect of week, *F*_(3, 21)_ = 3.54, *p* = .03, after applying the Greenhouse-Geiser correction to the degrees of freedom (Epsilon = 0.428). Inspection of the single degree of freedom polynomials revealed no linear component over weeks (*F*_(1, 7)_ = 0.46, *p* = .52) that would be consistent with systematic reductions in total step counts over the weeks of the study. Instead, the effects of time were described by significant quadratic (*F*_(1, 7)_ = 6.17, *p* = .04; 32.4% of the variance) and cubic polynomials (*F*_(1, 7)_ = 15.01, *p* = .006; 53.7% of the variance). This pattern of results does not suggest any clear reductions in step counts during the intervention that would indicate a replacement of typical stepping behavior with stair climbing (Fig. 2).

### Individual reports about the acceptability of the intervention

A full table of experimental participants’ responses during the post-intervention interview is available as supplementary material (Supplementary File). Concerning acceptability of the intervention, experimental participants enrolled in the study for physical activity and health reasons (7/8) and the majority reported resultant beneficial effects on health (6/8). Most found it easy or convenient to complete the stair climbing (7/8) and reported the breaks helpful to their work (6/8). Nonetheless, two questioned the sustainability of eight daily climbs. Unsurprisingly, the reported barriers were that it sometimes also interrupted their work (5/8) and did not always fit within a busy schedule (4/8). Nonetheless, seven said they would continue to climb stairs regularly, with the only dissenter, a retiring employee who would not have easy access to a sufficiently tall building, planning to walk more instead.

## Discussion

This pilot assessed the acceptability of a four-floor stair climb as an interruption to sitting during the working day. The experimental group logged 121 floors/week of the target of 160 floors. The magnitude of this response to the prompt suggests stair climbing is an acceptable interruption to sitting for employees. Participants completed four-floor stair climbing (ascent and descent) for about two minutes. There was preliminary evidence that increased stair use by the experimental group improved some biomarkers of lipid health and fasting blood glucose.

For office workers, 24.2 floors/day proved acceptable and sufficient to improve health (beneficial changes in LDL-C, TC, TC/HDL-C ratio, non-HDL-C and fasting blood glucose). Most employees said it was easy or convenient to climb when prompted and, for some, breaks were reported as helpful to work. Sometimes the prompt interrupted their work and stair use did not always fit within their busy schedules. The later was the reason why one experimental participant dropped out; frequent meetings elsewhere meant that she could not respond to prompts. Stair use interruptions would only be effective for employees who spend most of their time at the same desk and are unconstrained by long meetings elsewhere. Two participants questioned the sustainability of eight daily climbs, a reasonable query given the commitment required. Nonetheless, prompts are active environmental ‘nudges’ towards healthier choice [31]. They function by reminding individuals of prior intentions to choose healthy behaviours at a time and place where action can be taken [32–34]. They are post-decisional aids to health in that they require prior intentions for effectiveness [35]. Multi-component workplace interventions should target intentions to be more active and prompt the behaviour when it can be chosen [36]. An employee truly seeking to improve their health, i.e. reduce identified MetS risk, may be more persistent than the initially thought. Stair climbing is a plausible physical activity for most of the population because they already do it as part of their daily life [27]. Most participants joined the study for health reasons; the recruited sample was people having sedentary lifestyle at work. A prompting intervention might attract employees intending to increase stair climbing for its benefits.

From an organisational perspective, low cost programme of interrupted sitting would be preferable. It would need some support from the building management, in particular the IT department, but little else. Computers are ubiquitous for office staff. We were not allowed to recruit employees in finance as the management were concerned that the prompt might introduce errors by interrupting calculations. Employees with frequent meetings out of their office would miss many of the hourly prompts and may also be unsuited for the intervention. On the plus side, it seems likely that interruptions to the working day that total only 16 min would be acceptable to employers seeking to facilitate employees who wish to improve their health.

The current study involved eight ascents/day, with a target of 32 floors daily. There were no changes in the control group, consistent with Boreham et al. [28, 29]. For the experimental group in this pilot, there were significant improvements of TC, TC/HDL-C ratio, LDL-C, and non-HDL-C, termed ‘bad cholesterol’. Six ascents/day targeting 48 floors in Boreham et al. (2000) improved TC, TC/HDL-C ratio and HDL-C whereas five ascents/day targeting 40 floors in the group’s 2005 paper improved only LDL-C [28]. The improvements in lipoproteins with a four-floor climb, eight times/day appear similar to the eight-floor climbs in Boreham and colleagues’ research. In addition, fasting blood glucose was reduced. Participants here descended the stairs after their climb whereas in Boreham’s studies, an available lift adjacent to the stairs would be the likely means of descent after an eight-floor climb (Boreham, personal communication April, 2021). The preliminary data here suggest that more frequent climbs of a lower height have at least comparable effects to eight-floor climbs. This may be important to acceptability; lower climbs entail a shorter interruption during work and should be acceptable to employees and employers. Similar to the data here, a reduction of LDL-C was found in a 12-week intervention of 21 floors/day [37]. LDL-C is strongly associated with increased risk of coronary heart disease and reductions of LDL-C would reduce the risk [38].

Fasting blood glucose and 2 h plasma glucose for both groups were within the healthy range, < 5.6 mmol and < 7.8 mmol respectively [39]. Despite this, fasting blood glucose was reduced by walking up and down stairs at work in this study. Acute glycaemic control post-prandially was improved by walking up and down a single floor of stairs in sedentary, middle-aged men [40] and individuals with type 2 diabetes mellitus [41–44]. The descent component of stair use may be important. Descent improved glycaemic control more than ascent when the behaviours were formally compared in women living with obesity [45, 46]. The eccentric nature of exercise when descending may be an important bonus of an alternating ascent and descent protocol for disease risk [47]. Consistent with this, daily stair usage at home decreased MetS risk even after adjustment for age, sex, socioeconomic position, marital status, smoking, self-reported health and sports participation [47]. The preliminary data here suggest improvements in participants with healthy serum glucose. It seems likely that effects would be greater for diabetic and prediabetic individuals. Nonetheless, a recent alternating ascent and descent protocol at home improved the MetS risk factors of glucose, HDL and triglycerides in healthy females [27]. The preliminary data here need confirmation with a larger sample; Michael and co-workers tested 26 ascending and descending participants [27].

Costs for treating CVD in the EU and the US were estimated at 8% and 17% of total health care expenditure respectively [48, 49]. For diabetes, a major component of MetS risk, costs were estimated at 10% and 14% for the EU and the US respectively [50, 51]. Minimization of health care costs is a pervasive aim of governments. A recent extensive trial of sit-to-stand intervention, *Stand Up Victoria* replaced sitting at work with standing, with modest reductions in MetS risk and fasting glucose. To achieve this, Healy et al., (2017) installed sit-to-stand work stations ($400 each), had a liaison employee who linked with the team, a lecture to the management and brainstorming for the workplace [9]. Employees attended a workshop followed by 30 min, 1-to-1 interviews with a health coach who subsequently called each individual. This multi-component research intervention revealed what could be achieved with unlimited resources. Employers, however, think the cost of workplace interventions for population health should be funded by other agencies [52, 53]. Minimized cost is an agenda on which business and health care agree. This prompting intervention is low-cost for both. A scheduler, installed in Microsoft outlook, prompts individuals to use a workplace fixture to reduce disease risk. Brief interruptions to sitting are plausible for most, acceptable to employees and may facilitate work. Employers were willing to host low-cost prompting interventions for stair use but sceptical about their value [52]. This intervention can deliver stair use prompts directly to at-risk employees, i.e. those likely to respond. Improved health for employees, who also report benefits to work wellbeing, from 16 min of climbing/day offers a low-cost option which corporate workplace health might find attractive.

### Study strength and limitations

This study highlighted an alternative (i.e. stair climbing) to the common physical activities at work such as standing and walking, but with greater energy expenditure. Apart from that, this study was conducted in the employers’ context and work location. Thus, the findings represented the real world applicability and feasibility. This was also a cost effective intervention as the use of task scheduler and stairs required zero cost but able to improve health of the employees. However, this study had several limitations. It was an oversight not to measure waist circumference in the sample to better assess their MetS risk status. The baseline data for the behavioural measures before the start of the intervention was not obtained. These data would be beneficial to measure any behavioural change during the intervention. Stair use was not measured directly but instead recorded on a log sheet by the computer. Electronic tagging could remove this uncertainty about the dosage of the intervention. The study design was not a randomised control trial as we used the quasi experimental design. Volunteers who were willing to participate in this study and working in a building with four floors and above were placed under the experimental group. Participants working in the lower than four-floor building were in the control group. Furthermore, the small sample size of the study had reduced the statistical power in relation to the health biomarkers, thus did not detect other changes that possibly were practically significant in this study. Finally, there was also no follow up after the intervention ended to observe the sustainability of the stair climbing activity.

### Recommendations

The amount of stair use needs to be recorded directly and measuring behaviour using accelerometer or inclinometer would be beneficial in any future full-scale trial. While there were reductions in fasting glucose, there were no improvements in the MetS risk markers of HDL-C or TG in this pilot. Triglycerides were reduced in only one of five previous studies, the one with the largest sample [32, 34, 37, 42, 54]. For HDL-C, the pilot sample was 50% smaller than Boreham et al. (2000) [28]. Nonetheless, lower levels of HDL-C at baseline would have allowed an increase [c.f. Boreham et al., (2000) vs. Table 2]. Lack of effects on HDL-C might reflect variability in lipid concentrations as samples were collected within 24–48 h of the last stair climbing day; a 72-hour gap would have been preferable [54]. Alternatively, lipoproteins were affected by the meal consumed on the evening prior to the test. Any variation in the evening meals prior to the initial and post-intervention sessions would add noise to the data.

## Conclusion

Prompting interruptions while sitting at work substantially increased logged stair use in this pilot. Most interviewed participants found the interruptions acceptable and beneficial. All participants able to do so, reported that they would continue to climb more stairs in the near future. Eight weeks of stair ascending and descending improved some lipoproteins and fasting blood glucose. MetS risk factors of HDL-C and triglycerides were not changed. This simple intervention might provide a low-cost approach to disease risk reduction at work, acceptable to employees and employers alike.

## Electronic supplementary material

Below is the link to the electronic supplementary material.

Supplementary Material 1

## Data Availability

Data are available from the authors upon reasonable request and can be obtained from the corresponding author, Intan Suhana Munira Mat Azmi.
