# Supplementary material for: Desk based prompts to replace workplace sitting with stair climbing; a pilot study of acceptability, effects on behaviour and disease risk factors
Source: BMC Public Health. 2022 Oct 31;22:1985. doi: 10.1186/s12889-022-14393-1 (PMC9620615; doi:10.1186/s12889-022-14393-1)
Supplement: Supplementary file 1 — Supplementary Material 1 [file 12889_2022_14393_MOESM1_ESM.docx]

Supplementary File: Participant responses to the post intervention interview questions.

| **Items** | **Questions** | **Participants responses** |
| --- | --- | --- |
| 1. | Why did you volunteer for this study? | E01 – Enjoy participating and potential health benefit  E02 – To undertake regular exercise to get fitter  E03 – Try to get healthier  E04 – Want to exercise  E05 – To help and to get fitter and to see what difference does it make  E06 – To increase exercise and reduce weight  E07 – To test my ability & stamina  E08 – Helping a friend in research |
| 2. | Was the stair climbing intervention convenient/easy for you? | E01 – Yes easy, although going and climb the stairs every hour eight times a day is not sustainable  E02 – It was convenient as the stairs were easily accessible  E03 – It was moderate, not that easy  E04 – Yes easy  E05 – Easy, just the hour between climbing was too quick  E06 – Yes convenient, very accessible  E07 – In the beginning it was easy, but towards the end it became quite demanding  E08 – Yes |
| 3. | Did the intervention help or interrupt with your work? | E01 – Both, provide useful breaks but sometimes breaks were inconvenient  E02 – Did interrupt with my work some days, and on really  busy days with lots of meetings and difficult to fit in  E03 – Helped  E04 – Yes it helped  E05 – Sometimes it did interrupt  E06 – Helped, as not continuously sitting all day  E07 – It helped with my concentration and focus because it kept me awake, only when I was in hurry or needed to attend other matters it became demanding  E08 – Little bit of both, depending on my schedule |
| 4. | Are you going to continue climbing stairs regularly? Why? | E01 – Yes, the study made me realised how sedentary my office day is  E02 – No, will be retiring and don’t have access to many stairs but will walk more  E03 – Yes, will continue with 2 floors (to keep fit and get away from sitting in front of computer)  E04 – Yes, I have definitely felt fitter because of that  E05 – Yes, try to do it regularly  E06 – Yes, feel better  E07 – Yes, it becomes a preference now  E08 – Yes |
| 5. | Would you do this activity again? | E01 – Yes  E02 – Possibly, given the opportunity  E03 – Yes, if only 2 floors  E04 – Yes  E05 – Yes  E06 – Yes  E07 – Yes  E08 - Yes |
| 6. | Can you think of any benefits from this intervention? | E01 – Feel fitter, more alert & help with my concentration  E02 – I felt slightly fitter and it certainly made me think about being more active  E03 – To get away from the screen, and take a break from work  E04 – I realise that I do not exercise enough  E05 – Loss of weight, feel fitter  E06 – Yes, it makes me realise lack of exercise/walking I undertake  E07 – Yes, it increased my focus  E08 – Increased stamina |
| 7. | Can you think of any barriers to this intervention? | E01 – Not sustainable for long term, in some days it surely doesn’t happen  E02 – None  E03 – No  E04 – No  E05 – The clash when students are crowd  E06 – Volume of work, meetings, not always allow for it  E07 – Clashed with personal schedule (holiday & vacation or unexpected occasions)  E08 – During tight schedule (meeting, lab etc.) |
| 8. | What would you like to improve from this activity? | E01 – Nothing  E02 – Fitness level  E03– I wish if you can replace that horrible glucose drink with something else  E04 – Nothing  E05 – Fitness level  E06– None  E07 – Consistency  E08 – Motivation |
| 9. | What did your colleague say about it | E01 – They were interested, some thought a bit odd when they saw me climbing stairs.  E02 – They were interested  E03 – Influenced other colleagues to climb stairs  E04 – They seem interested to climb 2 flights of stairs hourly  E05 – Nothing  E06 – Found it useful and good to help with exercising  E07 – Requires great commitment and challenging  E08 – Interesting research |
